# Expression of cluster of differentiation 47 (CD47) and signal regulatory protein alpha (SIRPα) as prognostic biomarkers and potentially therapeutic targets in esophageal squamous cell carcinoma

**DOI:** 10.1007/s10388-025-01152-5

**Published:** 2025-09-10

**Authors:** Junpeng Li, Yohei Ozawa, Takeru Mozumi, Kaiyuan Jiang, Yusuke Taniyama, Chiaki Sato, Hiroshi Okamoto, Hirotaka Ishida, Naoto Ujiie, Shinobu Ohnuma, Michiaki Unno, Takashi Kamei

**Affiliations:** Department of Surgery, Tohoku University Graduate School of Medicine, 1-1 Seiryo-Machi, Aoba-Ku, Sendai, Miyagi Japan

**Keywords:** Esophageal squamous cell carcinoma, Immune checkpoint inhibitor, Signal regulatory protein alpha, Cluster of differentiation 47, Tumor biomarker

## Abstract

**Background:**

The cluster of differentiation 47 (CD47)-signal regulatory protein alpha (SIRPα) axis is a key regulator of innate immune surveillance, facilitating the neoplastic evasion of macrophage-mediated phagocytosis. Although this pathway has been implicated in tumor immune escape in multiple malignancies, its clinical and prognostic significance in esophageal squamous cell carcinoma (ESCC) remain to be fully elucidated.

**Methods:**

We retrospectively analyzed 100 patients who underwent esophagectomy for resectable ESCC. Immunohistochemical testing determined SIRPα expression in peritumoral immune infiltrates and CD47 expression in tumor and immune cells, while tumor proportion score (TPS) and combined positive score (CPS) were used to evaluate CD47 staining. Survival outcomes and correlations with clinicopathological factors were also analyzed.

**Results:**

Increased expression of SIRPα, CD47 CPS, and CD47 TPS was detected in 47, 50, and 47% of patients, respectively. Elevated SIRPα expression was significantly associated with decreased overall survival. Also increased CD47 CPS and TPS was significantly associated with decreased overall survival and relapse-free survival. CD47 CPS was identified as an independent prognostic indicator for overall survival in multivariate Cox regression analysis (hazard ratio [HR] = 3.89; 95% confidence interval [CI] = 1.57–9.61; *P* = 0.003). Patients with concurrent high expression of both SIRPα and CD47 CPS demonstrated the poorest survival outcomes.

**Conclusion:**

Overexpression of SIRPα and CD47, especially in tandem, is associated with poor clinical outcomes in ESCC, suggesting that the CD47-SIRPα axis may serve as a useful prognostic biomarker and a potential therapeutic target for newly immune checkpoint blockade in ESCC.

**Supplementary Information:**

The online version contains supplementary material available at 10.1007/s10388-025-01152-5.

## Introduction

Esophageal squamous cell carcinoma (ESCC) exhibits particularly high incidence and mortality rates in certain regions, such as East Asia and portions of Africa, with its global burden projected to increase further in the coming years [1]. Despite recent advances in traditional treatment modalities including surgery, radiotherapy, and chemotherapy, the 5-year survival rate for patients with ESCC remains low [2], largely in part owing to disease recurrence and metastasis [3].

Immunotherapy has emerged as a promising treatment strategy for a wide range of malignancies, with immune checkpoint inhibitors (ICIs) in particular garnering much attention. Multiple clinical trials have demonstrated the clinical efficacy of programmed death-1 (PD-1) inhibitors in ESCC in recent years [4–6]. However, despite these advances, the clinical effectiveness of currently used PD-1 inhibitors in the treatment of ESCC remains limited, due to multiple tumor immune evasion mechanisms. Among these, adaptive immune resistance and innate immune suppression are key factors contributing to the low response rate [7]. Therefore, the identification of novel immune checkpoint targets able to overcome resistance to treatment is crucial for improving treatment outcomes.

Signal regulatory protein alpha (SIRPα) is highly expressed in neurons and myeloid hematopoietic cells including macrophages, neutrophils, and dendrites [8]. Macrophages play a key role in phagocytosing target cells during innate immune responses. However, the interaction between SIRPα on macrophages and cluster of differentiation 47 (CD47) on target cells inhibits macrophage-mediated phagocytosis, forming the SIRPα-CD47 axis and serving as an innate immune checkpoint. This mechanism enables tumor cells to evade immune surveillance, making it a critical target for immunotherapy in the setting of malignancy [9]. In recent years, the CD47-SIRPα pathway has been shown to play a crucial role in immune evasion and tumor progression in various cancers, including laryngeal squamous cell carcinoma, non-small cell lung cancer, rectal cancer, and acute myeloid leukemia [10–13]. Building on these findings, therapeutic agents targeting the CD47–SIRPα axis—particularly anti-CD47 monoclonal antibodies—have shown promising antitumor activity in preclinical studies [14, 15]. Although these studies highlight the prominent role of the CD47-SIRPα pathway in inhibiting tumor cell phagocytosis and promoting tumor immune evasion, the specific role and immunoregulatory mechanisms of the CD47-SIRPα pathway in ESCC remain poorly understood.

This study investigated the association between the expression of the CD47-SIRPα axis, clinicopathological characteristics, and survival in patients with ESCC who have undergone esophagectomy; assessed its prognostic significance; and evaluated its potential therapeutic value as a target for modulating the CD47-SIRPα pathway.

## Materials and methods

### Case selection

This study included 100 patients with esophageal squamous cell carcinoma who underwent esophagectomy at Tohoku University Hospital between 2015 and 2021. None of the patients received preoperative radiation therapy or additional resection after endoscopic resection. The patient selection flowchart is shown in Supplementary Figure S1. The study protocol was approved by the Ethics Committee of the Tohoku University School of Medicine (Approval no. 2024-1-619). The need for informed consent was waived due to the retrospective nature of the study. Instead, an opt-out method was employed by disclosing the study information on the institutional website.

### Data collection and definition

Clinicopathological data were retrospectively reviewed by using medical records, and all of the included patients were pathologically diagnosed with esophageal cancer according to the 8th edition of the American Joint Committee on Cancer/Union for International Cancer Control TNM Staging [16]. For patients without recurrence or death, data were censored from the time of the last follow-up. Overall survival (OS) was defined as the interval from the date of surgery to that of the patient’s death or final follow-up, and relapse-free survival (RFS) was defined as the interval from the date of surgery to that of tumor recurrence, final follow-up for patients without recurrence, or death.

### Immunohistochemical staining

All specimens were fixed in 10% formalin for 24 to 48 h and subsequently prepared as formalin-fixed paraffin-embedded tissue blocks. Continuous 3-μm-thick sections were cut from the FFPE blocks using a microtome for immunohistochemical (IHC) staining. The slides were placed in a microwave (500W-20 min) for antigen retrieval. Tissue sections were subsequently incubated for 30 min at room temperature in a blocking solution of 10% rabbit serum and incubated overnight at 4 °C with each primary antibody. A Histofine Kit (Nichirei Bioscience, Tokyo, Japan) was used for the streptavidin–biotin amplification method. Antigen–antibody complex was visualized with 3,3′-diaminobenzidine (DAB) solution (1mM DAB, 50 mM Tris–HCl buffer (pH 7.6), and 0.006% H2O2) and counterstained with hematoxylin. For CD47 staining, a rabbit monoclonal antibody (clone, EPR21794; catalog no., ab218810; dilution, 1:2000; Abcam, Cambridge, UK) was used, while for SIRPα staining, a rabbit polyclonal antibody (catalog no., ab8120; dilution, 1:1000; Abcam, Cambridge, UK) was used. All procedures were conducted strictly in accordance with the manufacturers’ protocols to ensure the accuracy and reproducibility of the results.

### Evaluation of immunohistochemistry

**Fig. 1:**
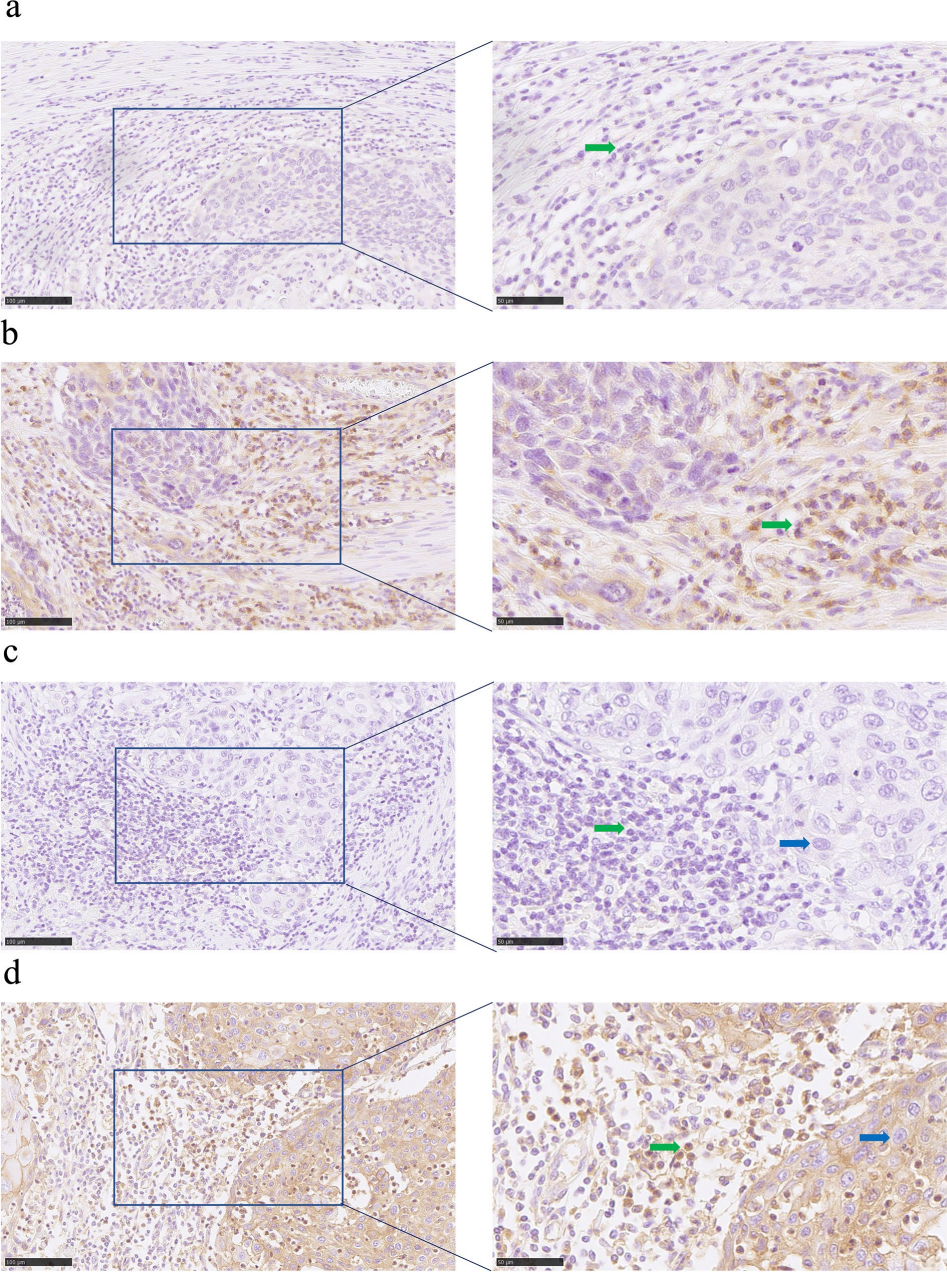
Representative immunohistochemical staining of signal regulatory protein alpha (SIRPα) and cluster of differentiation 47 (CD47) in surgical specimens. **a** Low SIRPα expression. **b** High SIRPα expression. **c** Low CD47 expression. **d** High CD47 expression. Blue arrows indicate tumor cells; green arrows indicate immune cells

SIRPα expression was evaluated by calculating the proportion of immune cells exhibiting membranous or cytoplasmic staining at the invasive margins of any tumor nests. SIRPα positivity was defined by a cutoff value of > 3%, as determined by receiver operating characteristic curve analysis (Supplementary Figure S2). CD47 expression was assessed using both the combined positive score (CPS) and tumor proportion score (TPS). CPS was defined as the percentage of CD47-positive tumor cells (partial or complete membranous staining of any intensity) and CD47-positive immune cells (membranous or cytoplasmic staining) relative to the total number of viable tumor cells, with values capped at 100. TPS was defined as the percentage of CD47-positive tumor cells relative to that of all viable tumor cells. CD47 positivity was defined as CPS or TPS ≥ 10%. IHC slides were evaluated blinded to the relevant clinical information. Representative IHC images of SIRPα and CD47 expression are shown in Fig. 1.

### Statistical analysis

Statistical analyses were performed using R software (version 4.3.2). Categorical variables were compared using Pearson’s chi-squared or Fisher’s exact test, as appropriate. The correlation between CD47 CPS, CD47 TPS, and SIRPα expression was evaluated using Spearman’s rank correlation coefficient. Survival curves were generated using the Kaplan–Meier method and compared using the log-rank test. Univariate and multivariate Cox proportional hazards models were used to evaluate independent prognostic factors for OS and RFS. Statistical significance was defined as a *P*-value < 0.05.

## Results

### Patient grouping and IHC characteristics

We performed IHC staining to evaluate the expression of CD47 and SIRPα in resected tumor tissues from 100 patients who underwent esophagectomy for ESCC. Representative images of SIRPα IHC staining are shown in Fig. 1a and b. SIRPα expression was predominantly localized to the cell membrane and cytoplasm of infiltrating immune cells, and based on IHC results, 47% of patients (*n* = 47) were classified into the high SIRPα expression group.

Figure 1c and d show representative CD47 staining image. CD47 is mainly localized on the cell membranes and within the cytoplasm of tumor cells, and in some immune cells. Based on CPS, 50% of patients (*n* = 50) were categorized as having high CD47 expression, while 47% of patients (*n* = 47) were classified into the high-expression group based on TPS.

A statistically significant positive correlation was observed between CD47 CPS and SIRPα expression levels (Supplementary Figure S3a), and between CD47 TPS and SIRPα expression levels (Supplementary Figure S3b).

### Baseline patient characteristics

The baseline patient characteristics and their relationship with SIRPα and CD47 expression levels are presented in Table 1. There were no significant differences in sex, age, median body mass index (BMI), smoking or drinking history, or clinical tumor stage between the high and low SIRPα expression groups. However, a significant difference was observed in the administration of neoadjuvant chemotherapy (NAC) between the high- and low-expression groups (*P* = 0.049). For CD47 CPS, there was a significant difference in NAC between the high- and low-expression groups (*P* = 0.011), and for CD47 TPS, significant differences were observed in clinical tumor stage (T) and NAC between the high- and low-expression groups (*P* = 0.046 and 0.049, respectively).

**Table 1:** Patients characteristics: SIRPα, CD47 CPS, and CD47 TPS in esophageal squamous cell carcinoma and their association with clinicopathological features

Variable	SIRPα		*P*-value	CD47 CPS		*P*-value	CD47 TPS		*P*-value
	High	Low		High	Low		High	Low	
	*N* = 47^1^	*N* = 53^1^		*N* = 50^1^	*N* = 50^1^		*N* = 47^1^	*N* = 53^1^	
*Age*			0.42^2^			0.82^2^			0.56^2^
< 75	37 (79%)	38 (72%)		37 (74%)	38 (76%)		34 (72%)	41 (77%)	
≥ 75	10 (21%)	15 (28%)		13 (26%)	12 (24%)		13 (28%)	12 (23%)	
*Sex*			0.12^2^			0.81^2^			0.58^2^
Male	34 (72%)	45 (85%)		39 (78%)	40 (80%)		36 (77%)	43 (81%)	
Female	13 (28%)	8 (15%)		11 (22%)	10 (20%)		11 (23%)	10 (19%)	
*Smoking*			0.42^2^			> 0.99^2^			0.78^2^
Yes	37 (79%)	45 (85%)		41 (82%)	41 (82%)		38 (81%)	44 (83%)	
No	10 (21%)	8 (15%)		9 (18%)	9 (18%)		9 (19%)	9 (17%)	
*Alcohol*			> 0.99^3^			> 0.99^3^			> 0.99^3^
Yes	43 (91%)	48 (91%)		46 (92%)	45 (90%)		43 (91%)	48 (91%)	
No	4 (8.5%)	5 (9.4%)		4 (8.0%)	5 (10%)		4 (8.5%)	5 (9.4%)	
*BMI*			0.79^2^			0.59^2^			0.79^2^
< 18	8 (17%)	8 (15%)		9 (18%)	7 (14%)		8 (17%)	8 (15%)	
> = 18	39 (83%)	45 (85%)		41 (82%)	43 (86%)		39 (83%)	45 (85%)	
*pT factor*			0.23^2^			0.16^2^			0.046^2^
T1-T2	21 (45%)	30 (57%)		22 (44%)	29 (58%)		19 (40%)	32 (60%)	
T3-T4	26 (55%)	23 (43%)		28 (56%)	21 (42%)		28 (60%)	21 (40%)	
*pN factor*			0.17^2^			0.31^2^			0.58^2^
N0-N1	32 (68%)	29 (55%)		33 (66%)	28 (56%)		30 (64%)	31 (58%)	
N2-N3	15 (32%)	24 (45%)		17 (34%)	22 (44%)		17 (36%)	22 (42%)	
*pStage*			0.08^2^			0.068^2^			0.20^2^
I–II	16 (34%)	10 (19%)		17 (34%)	9 (18%)		15 (32%)	11 (21%)	
III–IVa	31 (66%)	43 (81%)		33 (66%)	41 (82%)		32 (68%)	42 (79%)	
*NAC*			0.049^2^			0.011^2^			0.049^2^
Yes	31 (66%)	44 (83%)		32 (64%)	43 (86%)		31 (66%)	44 (83%)	
No	16 (34%)	9 (17%)		18 (36%)	7 (14%)		16 (34%)	9 (17%)	

^1^*n* (%), ^2^Pearson’s Chi-squared test, ^3^Fisher’s exact test

*SIRPα* signal regulatory protein alpha, *CD47* cluster of differentiation 47, *CPS* combined positive score, *TPS* tumor proportion score, *BMI* body mass index, *NAC* neoadjuvant chemotherapy

### Prognostic value of SIRPα, CD47 CPS, and CD47 TPS expression for OS and RFS

Kaplan–Meier survival analyses were conducted to evaluate the prognostic significance of SIRPα, CD47 CPS, and CD47 TPS for both OS and RFS, stratified by high and low expression levels.

**Fig. 2:**
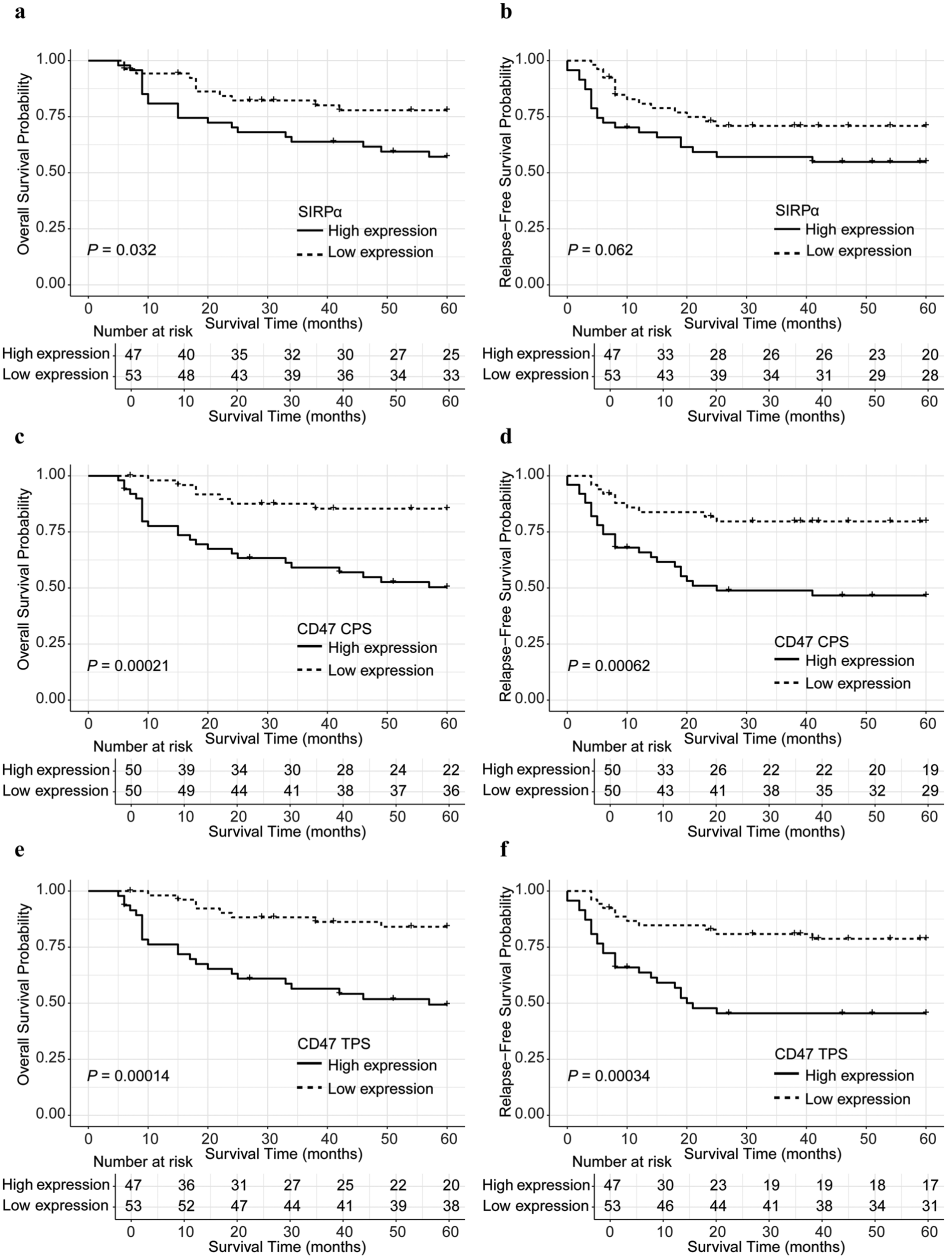
Kaplan–Meier survival curves according to the expression levels of SIRPα and CD47. Overall survival (OS) (**a**) and relapse-free survival (RFS) (**b**) stratified by SIRPα expression. OS (**c**) and RFS (**d**) stratified by CD47 combined positive score (CPS) expression. OS (**e**) and RFS (**f**) stratified by CD47 tumor proportion score (TPS) expression. Survival differences were compared using the log-rank test

Patients with high SIRPα expression exhibited significantly shorter 5-year OS (57.1%) than those with low expression (77.8%) (Fig. 2a; *P* = 0.032). In terms of 5-year RFS, the high SIRPα expression group also exhibited a lower survival rate (54.9% vs. 70.9%, Fig. 2b), but the difference did not reach statistical significance (*P* = 0.062).

High CD47 CPS expression was also significantly correlated with shorter 5-year OS (50.3%) in the high-expression group compared with 85.4% in the low-expression group (Fig. 2c; *P* = 0.00021). Elevated CD47 CPS expression was associated with worse 5-year RFS (46.7%) compared with 79.7% in the low-expression group (Fig. 2d; *P* = 0.00062).

A similar pattern was observed with CD47 TPS expression. High CD47 TPS levels were significantly associated with worse OS and RFS (Fig. 2e and f).

### Combined prognostic impact of SIRPα and CD47 expression

**Fig. 3:**
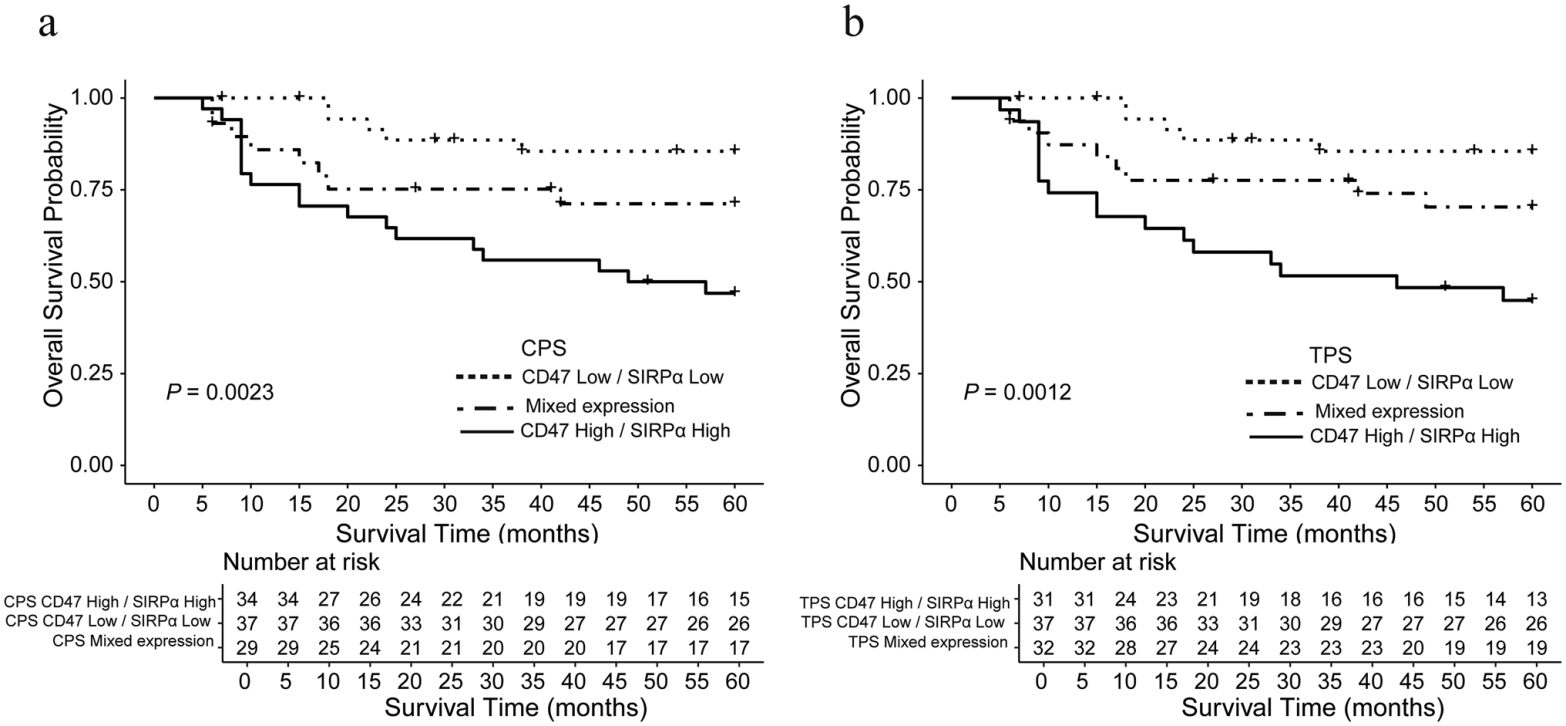
Kaplan–Meier survival analysis based on the combined expression levels of SIRPα and CD47. **a** Patients were categorized into three groups based on the combined expression levels of SIRPα and CD47 CPS: Low–Low group, patients with low expression of both markers; Mixed group, patients with high expression of either SIRPα or CD47; High–High group, patients with high expression of both SIRPα and CD47. (b) A similar analysis was performed using CD47 TPS instead of CPS. The same grouping criteria were applied

To further explore the combined prognostic impact of SIRPα and CD47, patients were stratified into three groups based on the expression status of both markers: low SIRPα/low CD47, mixed expression (low SIRPα/high CD47 or high SIRPα/low CD47), and high SIRPα/high CD47. Kaplan–Meier survival analysis revealed significant differences in OS among the three groups. Patients in the high SIRPα/high CD47 group exhibited the shortest median OS, indicating a markedly worse prognosis compared to the other two groups (Fig. 3).

### Univariate and multivariate Cox regression analyses of prognostic factors for OS

**Fig. 4:**
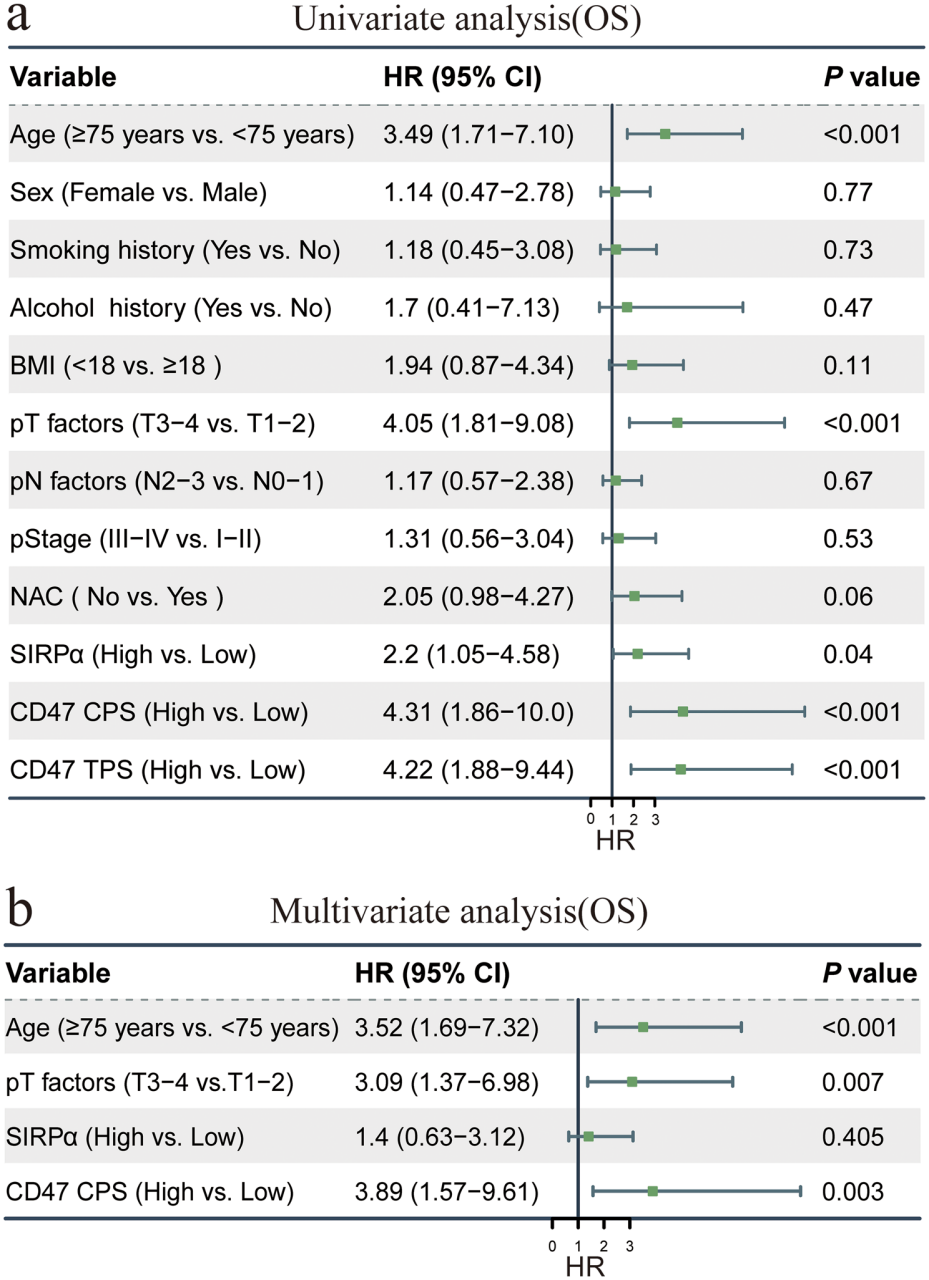
Univariate and multivariate Cox regression analyses for OS

Univariate Cox regression analyses revealed that several clinicopathological variables were significantly associated with OS (Fig. 4a). Patients aged over 75 years and those with advanced pathological T (pT) stage were association with worse OS. High expression of SIRPα, CD47 CPS, and CD47 TPS were significantly associated with reduced OS, with HRs of 2.2 (95% CI = 1.05–4.58; *P* = 0.04), 4.31 (95% CI =  1.86–10.0; *P* < 0.001), and 4.22 (95% CI = 1.88–9.44; *P* < 0.001), respectively.

To identify independent prognostic factors, variables with *P* < 0.05 in univariate analysis were included in the multivariate Cox regression model (Fig. 4b). This analysis confirmed that age ≥ 75 years and advanced pT stage remained independent predictors of poor OS. Of note, high CD47 CPS expression was also independently associated with worse OS (HR = 3.89; 95% CI = 1.57–9.61; P = 0.002).

### Exploratory analysis of CD47/SIRPα expression and ICI treatment outcomes

Among the 36 patients who experienced recurrence, 9 received immune checkpoint inhibitor therapy (nivolumab or pembrolizumab). RECIST data were available for eight of these patients. Supplementary Figure S4 presents the expression levels of SIRPα, CD47 CPS, and CD47 TPS, along with their corresponding RECIST outcomes. Due to the limited sample size, no statistical analyses were performed.

## Discussion

This study systematically investigated the prognostic significance of CD47 and SIRPα expression in patients with resectable ESCC, highlighting the role of the CD47-SIRPα axis as a potential mechanism of immune evasion and a target for immunotherapy. SIRPα is traditionally recognized for its role in interacting with CD47 to deliver a “do not eat me” signal, thereby inhibiting macrophage-mediated phagocytosis. However, SIRPα is not limited to macrophages—it is also expressed on various immune cells including dendritic cells, neutrophils, monocytes, and microglia, where it influences tumor immunity through multiple pathways [17]. Prior reports have identified high SIRPα expression as a marker of poor prognosis in several cancers [10–13]. Koga et al. [18] have reported the relationship between high SIRPα expression on both tumor and infiltrated stromal cells and poor prognosis among patients with ESCC. Given the broad distribution and functional relevance of SIRPα, this study further evaluated its expression in immune cells within the tumor microenvironment rather than in tumor cells. The findings of this study demonstrated that high SIRPα expression in infiltrating immune cells was significantly associated with worse survival outcomes, further reinforcing its role as a negative prognostic factor in patients with ESCC.

Additionally, CD47 overexpression has been reported in several malignancies and is often associated with a poor prognosis [19–21]. Especially, Wang et al. have reported that high expression of CD47 on tumor cells is a prognostic risk factor in ESCC [21], which was a similar approach to that used in TPS in this study. Therefore, we assessed CD47 expression not only tumor cells (TPS) but also combined with immune cells (CPS) in current study (TPS and CPS commonly utilized in the evaluation of immunotherapy biomarkers, including in PD-1/PD-L1 inhibitor studies approved in Japan [22–24]). The results showed that the prognostic impact of both was similar, and that CPS (and TPS, data not shown) was also detected as an independent prognostic factor, though the fact that the staining of the immune cells was extremely weak compared to that of the tumor cells. This finding suggests that CPS, along with TPS, may serve as a sensitive and comprehensive marker for evaluating CD47—an innate immune checkpoint molecule—in ESCC, similar to how PD-1 expression is assessed in T cells.

To the best of our knowledge, no previous studies have simultaneously evaluated CD47 and SIRPα expression in patients with ESCC. In this study, the positive correlation between CD47 and SIRPα expression observed in our study suggests a potential interaction between these two immune checkpoint molecules, which may contribute to immune evasion in ESCC. The use of NAC was more prevalent in the group with lower CD47 and SIRPα expression. Chemotherapy can remodel the tumor microenvironment and may lead to downregulation of CD47 expression [25], thereby potentially suppressing the “do not eat me” signal and enhancing macrophage-mediated phagocytosis of tumor cells although further investigation needed. Besides, patients with high CD47 and SIRPα expression had the worst survival outcomes among the three groups. These observations support the hypothesis that NAC may reduce CD47 and SIRPα expression in some patients by modulating tumor–immune interactions. This synergistic effect underscores the central importance of the CD47-SIRPα axis in shaping the immunosuppressive tumor microenvironment. These findings support a promising therapeutic strategy involving anti-CD47 and/or anti-SIRPα targeted therapies, which may enhance clinical benefits by restoring the innate immune response against tumors. Given their distinct mechanisms of action compared to existing immune checkpoint inhibitors such as anti–PD-1 antibodies, there is a theoretical basis for combination use. In addition, these therapies may be integrated with conventional treatments such as chemotherapy or radiotherapy, potentially expanding their applicability across various treatment regimens.

Beyond the inhibition of macrophage-mediated phagocytosis, recent studies have shown that blocking the CD47-SIRPα axis activates the cyclic GMP-AMP synthase—stimulator of interferon genes pathway in dendritic cells [26], enhances memory T cell responses [27], and improves NK cell cytotoxicity [28]. Moreover, CD47 blockade in effector T cells increases granzyme B expression, further augmenting Cytotoxic T lymphocytes mediated tumor killing [29]. Together, these findings suggest that the CD47-SIRPα axis may play a multifaceted role in the modulation of the tumor immune microenvironment in ESCC.

In addition, we performed an exploratory analysis of patients in our cohort who received immune checkpoint inhibitor therapy after recurrence, aiming to preliminarily assess the potential association between CD47/SIRPα expression and treatment outcomes. Given the small number of cases, no statistical analyses were performed, and the results are presented solely for descriptive purposes. Although this analysis does not allow definitive conclusions, it suggests that CD47/SIRPα expression levels may represent a potential biomarker for predicting responses to immunotherapy, warranting further validation in larger prospective studies.

This study had some limitations. First, this was a retrospective single-center study, which may limit the generalizability of the findings. Second, approximately 75% of the patients received neoadjuvant treatment in this study; therefore, potentially altering the tumor immune microenvironment and affecting the expression levels of SIRPα and CD47. Third, although we evaluated SIRPα expression in immune cells, the heterogeneity among different immune cell subsets was not fully explored; therefore, further research utilizing multiplex IHC or single-cell analysis is needed to clarify the role of SIRPα in specific immune cell populations. Finally, it has not been confirmed whether blocking the SIRP-CD47 axis actually activates the innate immune system; however, we have already begun work on this verification to confirm this immune activation.

In conclusion, this study demonstrated that the overexpression of CD47 and SIRPα, particularly in tandem, is significantly associated with poor prognoses among patients with resectable ESCC, with CD47 CPS identified as an independent prognostic factor. These findings may highlight the CD47-SIRPα axis as a key mechanism of immune evasion, showing its potential as both a prognostic biomarker and a novel therapeutic target in the treatment of ESCC.

## Supplementary Information

Below is the link to the electronic supplementary material.

Supplementary file1 (DOCX 4879 KB)

## Data Availability

The data that support the findings of this study are not openly available due to reasons of sensitivity; however, they are available from the corresponding author upon reasonable request.
