# Supplementary material for: Expression of cluster of differentiation 47 (CD47) and signal regulatory protein alpha (SIRPα) as prognostic biomarkers and potentially therapeutic targets in esophageal squamous cell carcinoma
Source: Esophagus. 2025 Sep 10;23(1):251–60. doi: 10.1007/s10388-025-01152-5 (PMC12832579; doi:10.1007/s10388-025-01152-5)
Supplement: Supplementary file 1 — Supplementary file1 (DOCX 4879 KB) [file 10388_2025_1152_MOESM1_ESM.docx]

**Supplementary materials**

**Supplementary Figure S1**

**
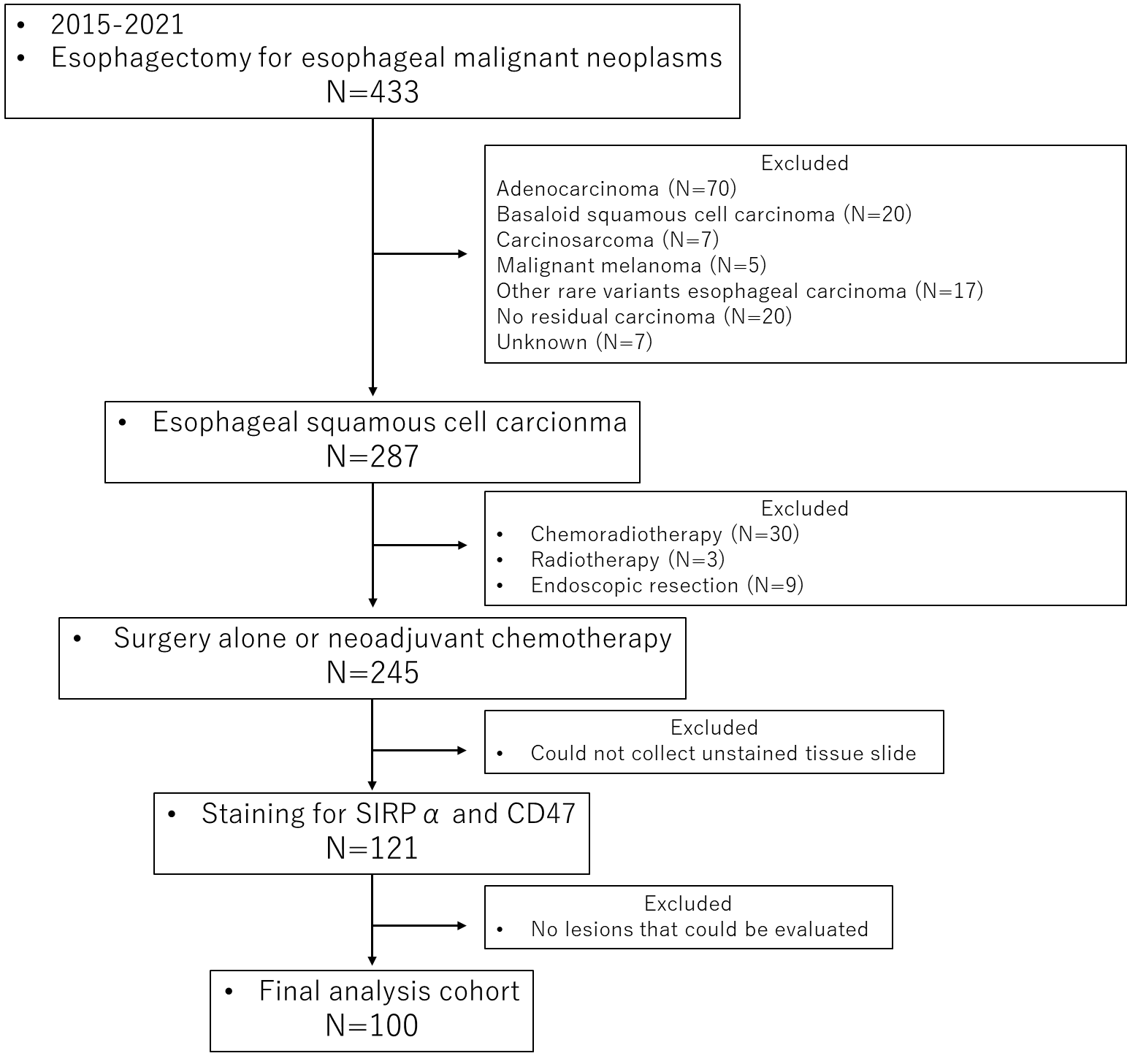
**

**Flowchart of the study population**

During the study period, 245 patients with esophageal squamous cell carcinoma underwent esophagectomy without initial treatment of irradiation therapy or additional resection after endoscopic resection. Unstained tissue slides were collected from tissue specimens for 121 cases including the deepest part of the tumor. After excluding cases in which the cancer lesions could not be evaluated, we analyzed data from 100 patients.

**Supplementary Figure S2**


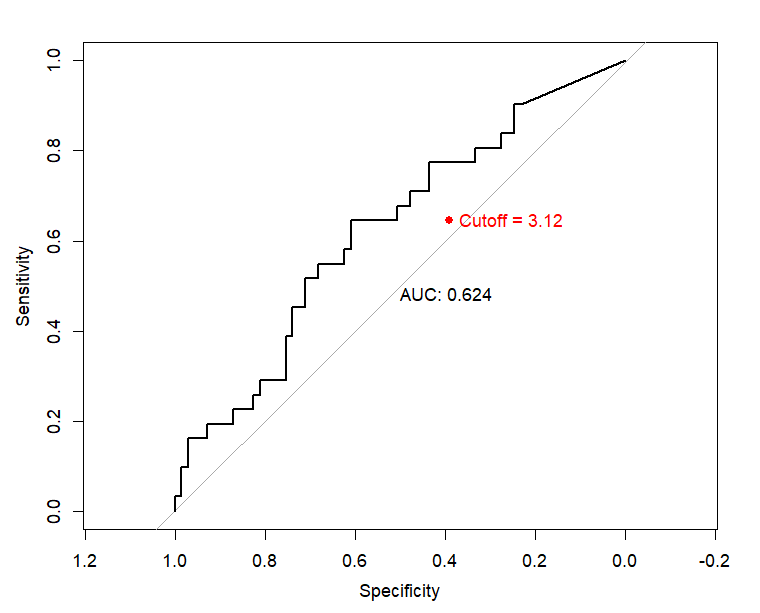


**ROC curve analysis to determine the optimal cutoff for SIRPα positivity.**
The ROC curve was generated to assess the diagnostic performance of SIRPα expression in immune cells at the invasive margin. The maximum Youden index was observed at 3%, which was used as the threshold in subsequent analyses.

**Supplementary Figure S3**

(a)


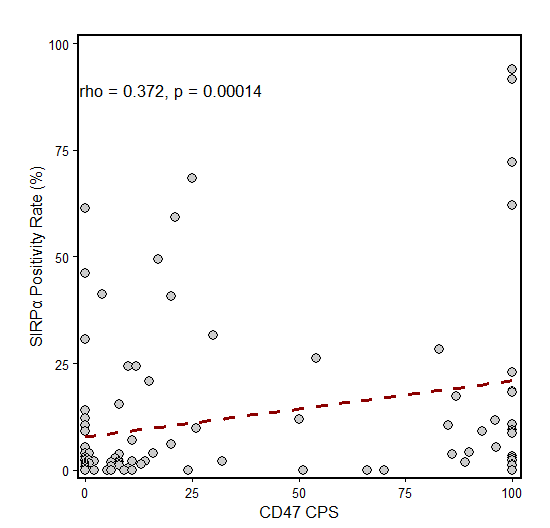


(b)


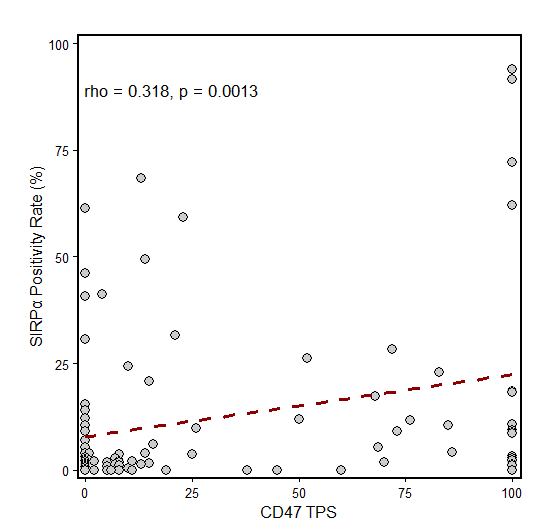


(a) Correlation between CD47 CPS and SIRPα positivity (Spearman’s correlation coefficient r = 0.372, P = 0.00014). (b) Correlation between CD47 TPS and SIRPα positivity (Spearman’s correlation coefficient r = 0.318, P = 0.0013).

**Supplementary Figure S4**


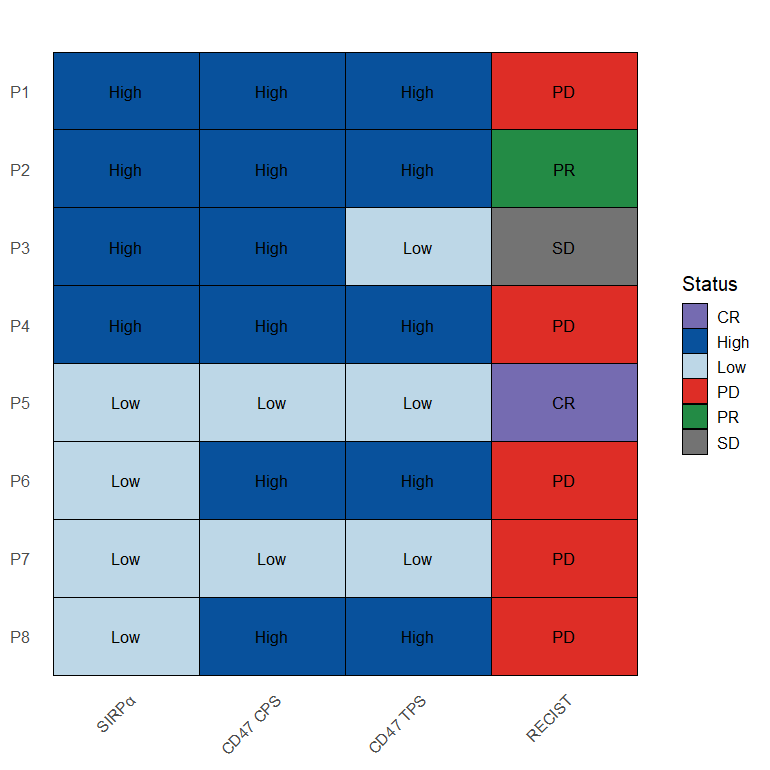


Expression patterns of SIRPα, CD47 CPS, and CD47 TPS in 8 recurrent patients who received immune checkpoint inhibitor therapy and had evaluable RECIST outcomes. Colors indicate high or low expression levels, while RECIST results are classified as complete response (CR), partial response (PR), stable disease (SD), or progressive disease (PD).
